# The Story of the Insane from Year to Year

**Published:** 1889-08-10

**Authors:** 


### The Story of the Insane from Year to Year.

Asylum Reports.

### SUFFOLK COUNTY ASYLUM AT WOODBRIDGE.

This is one of the oldest institutions for the insane in England. It contained at the end of 1888, 536 patients, being 16 in excess of the number for which accommodation is provided. The recovery rate stands at 40·76 per cent., and the death-rate at 15·69 per cent. The latter is unusually high and is doubtless accounted for by the prevalence of enteric fever and dysentery, 9 having died of the former, and 12 of the latter. Erysipelas accounts for 2 and phthisis for 13. The weekly cost is 9s. 5d. The superintendent's report is a very long one, extending to upwards of 20 pages. Dr. Eager says: "It will be well indeed if asylums under the new regime become recognised, to a greater extent than heretofore, as hospitals for the treatment of disease, rather than houses for the detention of those who are either too dangerous, or who, on account of being objectionable in many ways, are considered too troublesome to be taken care of in the workhouse or by their friends." We fail to see any good reason why a county asylum should not be both a hospital and an asylum, and we greatly doubt the policy of allowing the insane to be taken care of in workhouses. It rarely answers.

### CUMBERLAND AND WESTMORELAND.

The visitors give a short review of the asylum during the quarter of a century or so of its existence. In 1862 it was opened, and was intended for 200 patients. It was added to in 1866, and in 1876 steps were taken for its further enlargement. The following sentences are worthy of all acceptation: "In the opinion of your committee the consideration of the erection of a rate-supported training school for imbeciles cannot be long delayed. As to the advisability of such a measure no doubt exists. Imbecile children are out of place in a lunatic asylum, and yet, if allowed to grow up without training, as the majority do in these counties, they in time become the most expensive and troublesome patients met with in lunatic asylums." During 1888, 145 patients were admitted, 103 were discharged, 63 died, and on the last day of the year 554 were in residence. The recovery rate is very high, standing at 53 per cent. on the admissions. There were seven deaths from pneumonia, and five from phthisis; the percentage of deaths from all causes being 10·81 on the average number resident. Dr. Campbell has the following sensible remarks on restraint: "A feeble melancholic made such persistent and varied attempts to kill himself that I had to restrain him by mechanical means for a long period, and when in no other way was it possible for him to injure himself, he bit off his lower lip as far as he could reach it with his teeth. When such cases occur, the proper course is to do what is for the good of the patient and the safety of those around him." There cannot be a doubt of this, and a man who fails to resort to restraint, when restraint is the best treatment, fails in his duty to his patients. The weekly rate is a fraction under 8s.

### WORCESTER COUNTY ASYLUM.

At the close of 1858, the numbers in this asylum were 344, and during the last thirty years they have increased so much that there are now 839 in residence. During the past year 163 patients were admitted, 133 were discharged, and 68 died. Of the discharges, 42·5 per cent. were recovered: the average percentage since the opening of the asylum being 34·9. The deaths stand at 7·7 on the average number resident; the average death-rate in this asylum being 11·3. The actual cost of maintenance is only 6s. 11½d. per week per head. This is really too low, and it is to be hoped that next year's report will show a rise. Mechanical restraint has been used on one occasion only, and seclusion on seventy-seven occasions. A high percentage of the patients employ themselves.

### LEICESTER BOROUGH ASYLUM.

Until the opening of the new Derby Asylum, most of the Derby borough cases were received here, and with their departure the numbers fell considerably, so that at the end of the year there were 126 patients fewer than at the beginning, although 24 Leicester county cases had been admitted. The recoveries stand at 39·6 on the admissions, and the deaths at 8·3 on the average number resident. Phthisis caused death in five cases, and pneumonia in nine. The cost per head per week is 8s. 8¾d. The Commissioners in Lunacy recommend the erection of a new block for special dormitories and single-bedded rooms, and this work is to be carried out.

### BEDS., HERTS., AND HUNTS. ASYLUM.

This is generally known as the "Three Counties' Asylum," and is situated near Arlsey. The asylum was opened in 1860, and it then contained 422 patients. It finished the year 1888 with 1,032, but 60 of these did not belong to the "Three Counties." Nevertheless, there was an increase of 52 during the year, and the visitors think that some of these might be treated in workhouses "if proper accommodation existed.' The truth is that this proper accommodation never does exist in workhouses, and the only course which can be followed is to induce the friends of patients to take care of them at home in suitable cases, and to build for the increase which, making every allowance, is sure to happen. During the year 224 patients were admitted, 133 were discharged and 88 died; 33·5 per cent. were discharged recovered, the average here being 34·6. The death-rate was 8·5 against an average of 10·9. Dr. Swain remarks on the hopelessness of many of the cases admitted, and certainly there is some reason for the remark when it is seen than 29 were epileptics, 16 were paralytics, and 22 congenitally deficient. We notice that 13 of the deaths were owing to phthisis. The weekly charge to the unions was 8s.

### LANCASTER ASYLUM.

The numbers in this asylum have grown so much that there are now 1,996 in residence as compared with 1,583 at the close of 1887. It is so far fortunate for the Lancashire ratepayers that the increase is not wholly from their own county, but to a very considerable extent from other counties, not fewer than 300 having been received from Middlesex and Surrey. The recoveries stand at 34·69 per cent. on the admissions, and the deaths at 6·63 on the average number resident. Dr. Cassidy says that having regard to the cases admitted, these rates must be regarded as satisfactory, and we fully agree with him. We extract the following from his report: "There is nothing remarkable in the greater amount of general paralysis among men, but there is, I think, an increasing and, perhaps, significant, frequency of its occurrence among women. At one time a female paralytic was quite a rarity; now, according to my experience, it is increasing in frequency in both sexes, and is more rapidly fatal than it was twenty or even ten years ago. It is a disease of cities and large towns, and in this county nearly all our cases come from the great centres of population, even the Irish, who have immunity in Ireland, furnishing their contingency." Well, it is a terrible disease, and its absence from the sister isle is an inexplicable fact, like the absence of adders and toads. Perhaps immunity from all three is owing to St. Patrick. Dr. Cassidy's report is an extremely good one, and we regret being unable to quote more of it.

